# Characterisation of the chemical profiles of Brazilian and Andean morphotypes belonging to the *Anastrepha fraterculus* complex (Diptera, Tephritidae)

**DOI:** 10.3897/zookeys.540.9649

**Published:** 2015-11-26

**Authors:** Lucie Vaníčková, Radka Břízová, Antonio Pompeiano, Luana Lima Ferreira, Nathaly Costa de Aquino, Raphael de Farias Tavares, Laura D. Rodriguez, Adriana de Lima Mendonça, Nelson Augusto Canal, Ruth Rufino do Nascimento

**Affiliations:** 1 Laboratório de Ecologia Química, Instituto de Química e Biotecnologia, Universidade Federal de Alagoas, Av. Lourival de Melo Mota, s/n, Tabuleiro, CEP 57072-970, Maceió, AL, Brazil; 2 Institute of Organic Chemistry and Biochemistry ASCR, v.v.i., Flemingovo nám. 2, CZ-166 10 Prague 6, Czech Republic; 3 Institute of Chemical Technology in Prague, Technická 5, CZ-166 28 Prague 6, Czech Republic; 4 Laboratory of Plant Physiology, Centre of Agricultural Sciences, Federal University of Alagoas, Maceió, AL, Brazil; 5 Universidad del Tolima, Barrio Santa Helena Parte Alta, A.A. 546, CP 730006299, Ibague, Colombia

**Keywords:** Cryptic species, chemotaxonomy, GC×GC/MS, PCA

## Abstract

Fruit fly sexual behaviour is directly influenced by chemical and non-chemical cues that play important roles in reproductive isolation. The chemical profiles of pheromones and cuticular hydrocarbons (CHs) of eight fruit fly populations of the Andean, Brazilian-1 and Brazilian-3 morphotypes of the *Anastrepha fraterculus* cryptic species complex originating from Colombia (four populations) and Brazil (four populations) were analysed using two-dimensional gas chromatography with mass spectrometric detection. The resulting chemical diversity data were studied using principal component analyses. Andean morphotypes could be discriminated from the Brazilian-1 and Brazilian-3 morphotypes by means of male-borne pheromones and/or male and female CH profiles. The Brazilian-1 and Brazilian-3 morphotypes were found to be monophyletic. The use of chemical profiles as species- and sex-specific signatures for cryptic species separations is discussed.

## Introduction

Communication is a crucial process for both intra- and interspecific interactions (Kroiss 2008). Chemical signals are probably the oldest form of communication in living organisms, and insects constitute one group of animals that rely heavily on chemical communication signals (Levine and Millar 2009). Unique messages are created by varying the structure of the chemicals that comprise a message and by combining these chemicals in blends with different ratios (Jallon 1984, Millar 2000). Over time, the components of the messages have been tuned up by natural selection according to function within the context in which they evolved (Levine and Millar 2009, Vaníčková 2012).

The South American fruit fly, *Anastrepha fraterculus* (Wiedemann 1830) (Diptera: Tephritidae) constitutes a cryptic species complex with different degrees of pre- and postzygotic isolation (reviewed by Vaníčková et al. 2015a). Eight taxonomically distinct morphotypes have been recognized thus far; these are the Andean, Brazilian-1, Brazilian-2, Brazilian-3, Ecuadorian, Mexican, Peruvian and Venezuelan morphotypes (Hernández-Ortiz et al. 2004, 2012, 2015). Recent studies have shown that the Andean, Brazilian-1, Brazilian-3, Mexican, and Peruvian morphotypes have varying degrees of sexual incompatibility (Vera et al. 2006, Cáceres et al. 2009, Segura et al. 2011, Rull et al. 2013, Devescovi et al. 2014). *Anastrepha fraterculus* exhibits a complex mating system, which involves auditory, visual and chemical signals (Morgante et al. 1983, Aluja 1994). Males of this species form leks on host or non-host trees to release volatile compounds, which serve at first to attract other males and subsequently to attract conspecific females. These chemical mixtures therefore act as aggregation as well as sexual pheromones. Differences in the *Anastrepha fraterculus* male-borne volatiles produced by flies from geographically distinct populations from Argentina, Brazil and Peru were previously described (Lima et al. 2001, Cáceres et al. 2009, Břízová et al. 2013, Lima-Mendonça et al. 2014). Recently, chemical and electrophysiological analyses have shown that six compounds of the volatile mixture produced by males of *Anastrepha fraterculus* from northeastern Brazil are antennaly active in conspecific females (Milet-Pinheiro et al. 2015). The attractiveness of conspecific females in laboratory and semi-field bioassays for a synthetic mixture of the six components to conspecific females is similar to the attractiveness of male headspace samples (Milet-Pinheiro et al. 2015).

It is thought that tephritid fruit flies use contact pheromones in the last phase of courtship when a female briefly touches a male with its proboscis or front legs (Morgante et al. 1980, Vaníčková et al. 2014, Vaníčková et al. 2015b). Contact or short-range pheromones are relatively non-volatile, nonpolar compounds called cuticular hydrocarbons (CHs), which are synthesized from fatty acid precursors and deposited on an insect’s cuticle. Essentially, they serve as protection against desiccation. They also play a crucial role in communication and sex, species, or reproductive status recognition (Blomquist and Bagnères 2010, Kather and Martin 2012). Due to their species-specificity, CH profiles are widely used for cryptic species determination, as documented in diverse insect families such as Termitoidae (Haverty et al. 2000), Miridae (Gemeno et al. 2012), Tephritidae (Vaníčková et al. 2014, Vaníčková et al. in press), Drosophilidae (Oliveira et al. 2011, Jennings et al. 2014), and many others.

In *Anastrepha fraterculus* age- and sex-dependent CHs production has been investigated in one Argentinean population (Vaníčková 2012, Vaníčková et al. 2012b). Males of *Anastrepha fraterculus* from this population produce a set of unsaturated CHs, not present in female body washes, suggesting these compounds may function as contact pheromones in chemical communication. A recent study on intraspecific variation of male CH profiles from six populations of the *Anastrepha fraterculus* cryptic species complex, ranging from Argentina to Mexico, has shown that the chemical signatures are specific to the putative species and therefore may be used for identification of particular morphotypes within the complex (Vaníčková et al. in press). In the same study, female CH profiles of Brazilian-1, Peruvian and Mexican morphotypes revealed qualitative and quantitative differences in CH composition. Nevertheless, a detailed study using more populations of the same *Anastrepha fraterculus* morphotype from different regions is necessary for further evaluation of the male pheromone and CH profiles as potential chemotaxonomic markers in species differentiation. Furthermore, information on the variability of female CH profiles within putative species of the *Anastrepha fraterculus* complex needs to be evaluated.

The present work aims to (i) clarify differences in the composition of male-borne volatiles among eight different populations belonging to three morphotypes (Andean, Brazilian-1 and Brazilian-3) of the *Anastrepha fraterculus* cryptic species complex; (ii) evaluate the potential use of male CH profiles for *Anastrepha fraterculus* identification of the Andean, Brazilian-1 and 3 morphotypes; (iii) investigate divergence in female CH profiles of the Andean and Brazilian morphotypes.

## Methods

### Insects

Eight laboratory populations, previously analysed for the morphotype identification by Hernández-Ortiz et al. (2004, 2012, 2014 unpublished data), originated from unique collections (for more detail see Table 1). After eclosion, the insects were separated by sex and put into plastic chambers (30 × 20.5 × 16 cm). The flies were fed an artificial diet (Sobrinho et al. 2009). Brazilian morphotypes were reared in the Chemical Ecology Laboratory at the Universidade Federal de Alagoas (Maceio, Brazil). The temperature of the insectarium was 25 °C, relative humidity was 60%, and the photoperiod was 14:10 light:dark. The Andean morphotypes were kept in the entomological laboratories at the Universidad del Tolima (Ibague, Colombia). They were reared at 22 °C; relative humidity was 70%, and the photoperiod was 12:12 light:dark.

**Table 1.** Geographical location of the sampled populations of *Anastrepha fraterculus* in Brazil and Colombia and the analyses of their cuticular hydrocarbons (CH) and male-borne pheromones.

Country, State	Population	Code	Alt. [m]	Latitude	Longitude	Morphotype^†^	CH analyses	Pheromone analyses
Brazil, RS	Pelotas^‡^	PEL	7	29°28.19'S	50°37.03'W	Brazilian-1	+	+
Brazil, RS	Bento Gonçalves^‡^	BEN	690	29°17.08'S	51°51.89'W	Brazilian-1	+	+
Brazil, SC	São Joaquim^§^	SAO	1360	28°17.38'S	49°55.54'W	Brazilian-1	+	+
Brazil, AL	Alagoas^|^	AL	16	10°08.01'S	36°10.34'W	Brazilian-3	+	+
Colombia	Ibague^¶^	IBA	1580	04°26.20'N	75°13.55'W	Andean	n.a.	+
Colombia	Sibundoy^¶^	SIB	2136	01°20.33'N	76°91.92'W	Andean	+	+
Colombia	Duitama^¶^	DUI	2569	05°49.50'N	73°04.49'W	Andean	+	+
Colombia	Cachipay^¶^	CAC	1850	04°45.27'N	74°23.02'W	Andean	+	n.a.

† Morphotypes were identified by Dr. Hernández-Ortiz.

Institutions and laboratories which provided the particular populations:

‡ EMBRAPA-Empresa Brasileira de Pesquisa Agropecuária, Brazil;

§ UFB- Universidade Federal de Bahia, Brazil;

| UFAL-Laboratory of Chemical Ecology at Universidade Federal de Alagoas, Brazil;

¶ UT-Universidad del Tolima, Colombia.

n.a. – samples were not avaliable for the analyses.

### Male-borne volatile collection

For the male-volatile collection following populations were available: three Colombian (Duitama, Ibague, Sibundoy) and four Brazilian (Alagoas, Bento Gonçalves, Pelotas, São Joaquim) (Table 1). To obtain male-borne volatiles, procedures described by Břízová et al. (2013) and Milet-Pinheiro et al. (2015) were used. Groups of 20 sexually mature virgin males of *Anastrepha fraterculus* were placed in a glass desiccator (180 mm high; 200 mm diameter), and volatiles were collected using dynamic headspace methods. The inlet of the desiccator was modified by the addition of an inlet tube containing SuperQ® (100 mg; Chromapack) to adsorb the released volatiles. The volatiles were then collected using an air pump (Resun® AC 2600) coupled to a flow meter (Supelco®) and absorbed on the SuperQ® filter. The air flow through the filter was 500 mL min^-1^ for 24 h. The volatiles trapped in the filter were then eluted with 500 µL of redistilled trace analysis grade hexane (Sigma-Aldrich, Brazil). The samples were stored in 2 mL vials, which were kept in the freezer (-5 °C) until chemical analyses. Ten replicate samples of volatiles were collected for each of the study populations.

### Extraction of cuticular hydrocarbons

For the extraction of CHs following populations were available: three Colombian (Duitama, Cachipay, Sibundoy) and four Brazilian (Alagoas, Bento Gonçalves, Pelotas, São Joaquim) (Table 1). CHs of 20-days-old virgin males (*N* = 10) and females (*N* = 10) were extracted from the study populations following previously described methods (Vaníčková et al. 2012b, Vaníčková et al. 2014, Vaníčková et al. in press). 1-Bromdecane (Sigma-Aldrich, Czech Republic) was added as an internal standard for quantification (10 ng per 1 µL of the extract). Each extract was concentrated to approximately 100 µL under a constant flow of nitrogen and stored in a freezer (-5 °C) until analysis.

### Chemical analyses

Two-dimensional gas chromatography with time-of-flight mass spectrometric detection (GC×GC/MS) was used for the quantification and identification of male-borne volatiles and CH profiles. Identical conditions were used for the analyses of all samples and for chromatographic data evaluation, as described in previous studies on *Anastrepha fraterculus* male pheromones (Břízová et al. 2013, Milet-Pinheiro et al. 2015) and CHs (Vaníčková 2012, Vaníčková et al. 2012b, Vaníčková et al. in press). Standards of *n*-alkane (C_8_–C_38_; Sigma-Aldrich, Czech Republic) were co-injected with authentic samples to determine the retention indices (*RI*) of the analytes. Compounds were identified by comparison of their mass spectra fragmentation patterns, *RI* and authentic synthetic standards when available (Van Den Dool and Kratz 1963, Carlson and Yocom 1986, Vaníčková 2012, Vaníčková et al. 2012a, Břízová et al. 2013, Milet-Pinheiro et al. 2015, Vaníčková et al. 2014, Vaníčková et al. in press). Detailed chemical identification of the CHs has been published previously (Vaníčková et al. 2012b, Vaníčková et al. in press).

### Chemicals

Except for (*S,S*)-(-)-epianastrephin, which was provided by Prof. Jim Nation (University of Florida, Gainesville, USA), all chemicals were purchased either from Sigma-Aldrich, Brazil [α-pinene, limonene, (*Z*)-3-nonen-1-ol] or from Penta, USA [(*E*, *Z*)-3,6-nonadien-1-ol]. Chemicals were > 95% pure, based on the results from capillary gas chromatography.

### Statistics

The relative peak areas of seven male-borne volatile compounds and forty-eight male and female CHs (as identified by the GC×GC/MS in the deconvoluted total-ion chromatogram mode) were calculated for each replicate of the study populations.

The differences in the chemical composition of the samples from study populations were analysed by principal component analysis (PCA). Prior to PCA, peak areas were subjected to logarithmic transformation; intraspecific scaling was performed by dividing each species score by its standard deviation; the data were centred by species’ scores. In PCA analyses, hierarchical clustering based on Pearson correlation showed that populations with similar chemical profiles cluster together.

A heat map was used to visualize male-borne volatiles organized as matrices. The heat map performed two actions on a matrix. First, it reordered the rows and columns so that rows and columns with similar profiles were closer to one another, causing these profiles to be more visible to the eye. Second, each entry in the data matrix was displayed as a colour, making it possible to view the patterns graphically. Dendrograms were created using correlation-based distances and the Ward method of agglomeration was used in the present analysis (Key 2012). All computations were performed with R 3.1.2 (R Core Team 2014), and the R packages *FactoMineR* (Husson et al. 2015), and *gplots* (Warnes et al. 2015) were used.

## Results

### Male-borne volatiles

**Figure 1.**
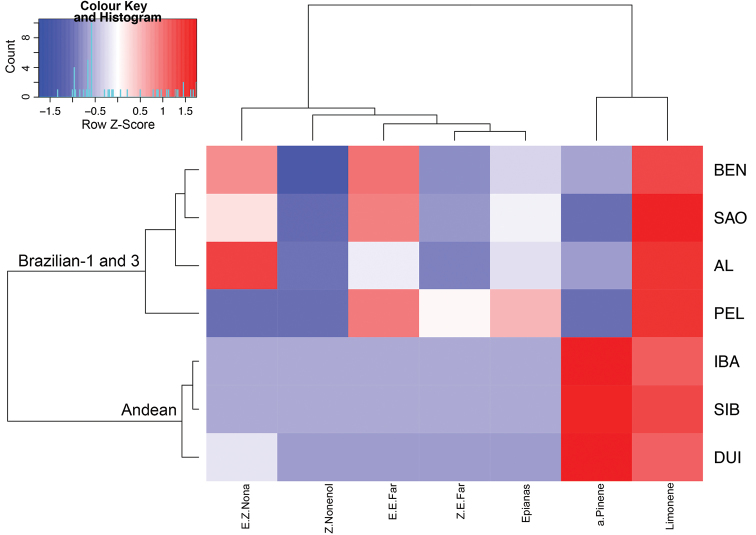
Heat map of seven male-borne volatiles (columns) identified by GC×GC/MS analyses in seven populations (rows) of the *Anastrepha fraterculus* cryptic species complex. The dendrograms were created using correlation-based distances and the Ward method of hierarchical clustering (*P* < 0.05). Key: AL – Alagoas, AL, Brazil; BEN – Bento Gonçalves, RS, Brazil; DUI – Duitama, Colombia; IBA – Ibague, Colombia; PEL – Pelotas, RS, Brazil; SAO – São Joaquim, SC, Brazil; SIB – Sibundoy, Colombia. Epianas – Epianastrephin; Z.E.Far – (*Z*, *E*)-α-farnesene; E.E.Far – (*E*, *E*)-α-farnesene; Z.Nonenol – (*Z*)-3-nonen-1-ol; E.Z.Nona – (*E*, *Z*)-3,6-nonadien-1-ol; a.Pinene – α-pinene.

Significant quantitative differences (*P* < 0.05) were found in the male-borne volatiles, namely α-pinene, limonene, (*Z*)-3-nonen-1-ol, (*E*, *Z*)-3,6-nonadien-1-ol, (*Z*, *E*)-α-farnesene, (*E*, *E*)-α-farnesene and epianastrephin, among the investigated populations of *Anastrepha fraterculus*. A heat map was constructed to visualize the relative proportions of the seven volatiles in each of the populations (Figure 1). Colombian populations (DUI, IBA, SIB) have similar proportions of the seven compounds, whereas the Brazilian populations (AL, BEN, PEL, SAO) have diverse volatile profiles. In the compound dendrogram, monoterpenes (α-pinene, limonene) formed one cluster, while sesquiterpenes [(*Z*, *E*)-, (*E*, *E*)-α-farnesenes], unsaturated alcohols [(*Z*)-3-nonen-1-ol, (*E*, *Z*)-3,6-nonadien-1-ol] and a lactone (epianastrephin) grouped in a second cluster. The most abundant volatile among all the populations was limonene. (*Z*)-3-Nonen-1-ol was the least abundant compound in the study strains. α-Pinene was more abundant in the Andean morphotype (DUI, IBA, SIB) than in the Brazilian morphotypes (AL, BEN, PEL, SAO). The relative proportion of (*E*, *Z*)-3,6-nonadien-1-ol varied the most between populations.

**Figure 2.**
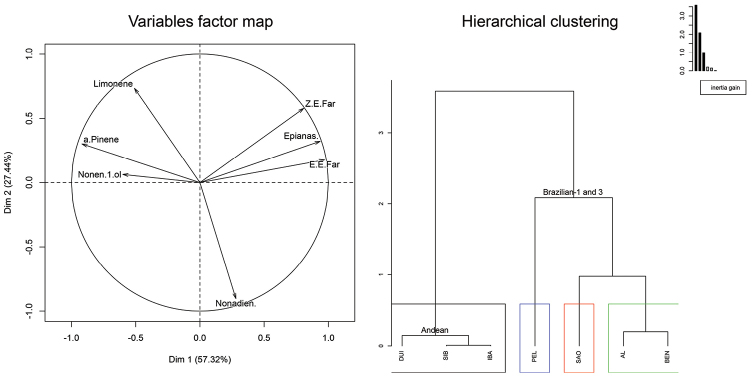
Principal component analyses (PCA) of transformed GC×GC/MS data of seven male-borne volatiles produced by groups of 20 sexually mature individuals from seven populations of the *Anastrepha fraterculus* cryptic species complex. Variables factor map represents projection of variables on the plane defined by the first two principal components. Hierarchical clustering is score plot describing the populations and their clustering. Key: AL – Alagoas, AL, Brazil; BEN – Bento Gonçalves, RS, Brazil; DUI – Duitama, Colombia; IBA – Ibague, Colombia; PEL – Pelotas, RS, Brazil; SAO – São Joaquim, SC, Brazil; SIB – Sibundoy, Colombia. Epianas. – Epianastrephin; Z.E.Far – (*Z*, *E*)-α-farnesene; E.E.Far – (*E*, *E*)-α-farnesene; Nonen.1.ol – (*Z*)-3-nonen-1-ol; Nonadien. – (*E*, *Z*)-3,6-nonadien-1-ol; a.Pinene – α-pinene. Colored boxes indicate particular clusters.

The PCA analyses of GC×GC/MS data showed the Andean morphotype formed one cluster while Brazilian-1 and Brazilian-3 morphotypes formed another cluster (Figure 2, Hierarchical clustering). The first two dimensions represented 85% of the total variance. The monoterpenes α-pinene and limonene and (*Z*)-3-nonen-1-ol were specific to the Andean morphotype, whereas sesquiterpenes (*Z*, *E*)- and (*E*, *E*)- α-farnesene, the lactone epianastrephin and (*E*, *Z*)-3,6-nonadien-1-ol were typical for the Brazilian morphotypes (Figure 2, Variables factor map). The three populations representing the Andean morphotype (DUI, IBA, SIB) grouped together forming the first cluster (Figure 2, Hierarchical clustering) whereas the second cluster was formed by the Brazilian populations representing the Brazilian-1 (BEN, PEL, SAO) and Brazilian-3 (AL) morphotypes.

### Male CH profiles

**Figure 3.**
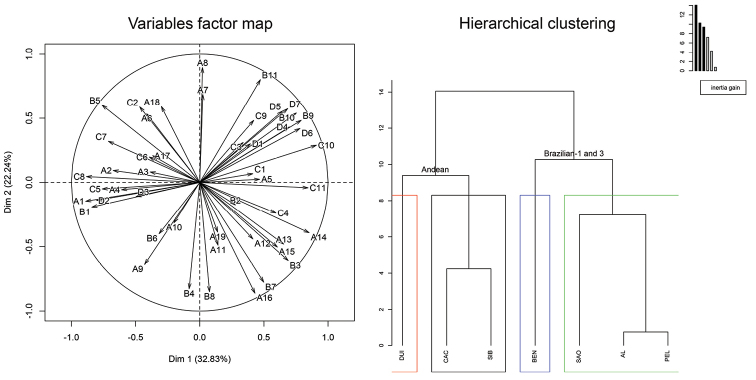
Principal component analyses (PCA) of transformed GC×GC/MS data of 48 male CHs from seven populations of the *Anastrepha fraterculus* cryptic species complex. Variables factor map represents projection of variables on the plane defined by the first two principal components. Hierarchical clustering is score plot describing the populations and their clustering. Key: AL – Alagoas, AL, Brazil; BEN – Bento Gonçalves, RS, Brazil; CAC – Cachipay, Colombia; DUI – Duitama, Colombia; PEL – Pelotas, RS, Brazil; SAO – São Joaquim, SC, Brazil; SIB – Sibundoy, Colombia. A1–19 – *n*-alkanes; B1–11 – methylbranched hydrocarbons; C1–11 – alkenes; D1–7 – alkadienes. Colored boxes indicate particular clusters.

Forty-eight male CHs, including 19 linear *n*-alkanes (A1-19), 11 methylbranched alkanes (B1-11), 11 alkenes (C1-11), and 7 alkadienes (D1-7), were evaluated by PCA for the possible use in the identification of Andean (CAC, DUI, SIB), Brazilian-1 (BEN, PEL, SAO) and Brazilian-3 (AL) morphotypes (Figure 3). The first two PCA dimensions accounted for 55% of the total variance. The populations clustered in two main groups, and each group was composed of two clusters. Clusters one and two were formed by Andean morphotype populations (DUI and CAC, SIB, respectively), whereas clusters three and four consisted of Brazilian morphotypes populations (BEN and AL, PEL, SAO; Figure 3, Hierarchical clustering). Linear *n*-alkanes (*n*-nonacosane A14, *n*-hentriacontane A16), methylbranched hydrocarbons (3-methylheptacosane B3, methylheptatriacontane B11) and 11-tritriacontene (C10) were responsible for the formation of the Brazilian morphotype clusters (AL, BEN, PEL, SAO), whereas *n*-dodecane (A1) and the mix of odd methylbranched hydrocarbons (9-/11-/13-methylnonacosane B5) were characteristic for the Andean group (Figure 3, Variables factor map, Suppl. material 1).

### Female CH profiles

**Figure 4.**
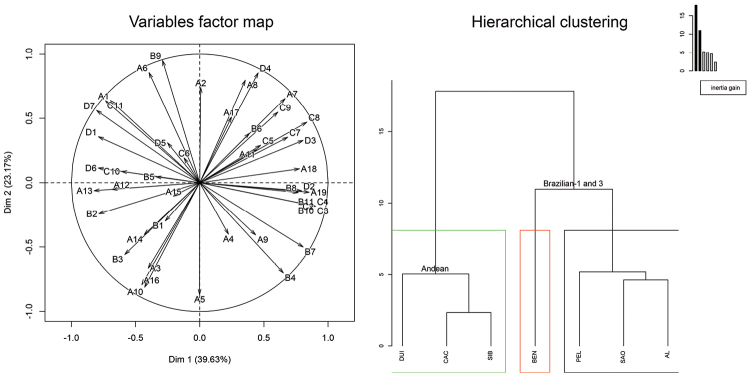
Principal component analyses (PCA) of transformed GC×GC/MS data of 48 female CHs from seven populations of the *Anastrepha fraterculus* cryptic species complex. (Variables factor map) projection of variables on the plane defined by the first two principal components. (Hierarchical clustering) score plot describing the populations and their clustering. Key: AL – Alagoas, AL, Brazil; BEN – Bento Gonçalves, RS, Brazil; CAC – Cachipay, Colombia; DUI – Duitama, Colombia; PEL – Pelotas, RS, Brazil; SAO – São Joaquim, SC, Brazil; SIB – Sibundoy, Colombia. A1–19 – *n*-alkanes; B1–11 – methylbranched hydrocarbons; C1–11 – alkenes; D1–7 – alkadienes. Colored boxes indicate particular clusters.

Female CH profiles from study populations consisted of 48 saturated and unsaturated compounds with chain lengths ranging from 12-38 carbons. In female body washes, unsaturated male-specific CHs were absent, namely 7-heneicosene, 7-docosene, 7-tricosene and 7-pentacosene. In the PCA analyses the populations segregated into two main groups (Figure 4, Hierarchical clustering). The three Colombian populations (CAC, DUI, SIB) grouped together forming one cluster. The second cluster was formed by the four Brazilian populations (AL, BEN, PEL, SAO) (Figure 4, Hierarchical clustering). The compounds responsible for this separation were *n*-docosane (A7), methylbranched hydrocarbons (2-methyloctacosane B4, 3-methylnonacosane B7) and unsaturated CHs (hentriacontene C8, tetratriacontadiene D4), which were specific to the Brazilian morphotypes. The Andean morphotype (CAC, DUI, SIB) was defined by the presence of linear compounds (*n*-dodecane A1, *n*-pentacosane A10, *n*-hentriacontane A16), a mix of methylbranched CHs (9-/11-/13-methylhentriacontane B9) and heptatriacontadiene (D7) (Figure 4, Variables factor map, Suppl. material 1).

## Discussion

Chemical profiles of *Anastrepha fraterculus* varied quantitatively among populations from diverse regions of South America. To some extent, the chemical profiles showed comparable patterns among populations belonging to the same morphotype. Comparison of the chemical profiles of the Andean and two Brazilian morphotypes showed that the more geographically distant the morphotypes are, the more diverse their pheromone and CH profiles are. Nevertheless, this trend was not observed between the Brazilian-1 and Brazilian-3 morphotypes. Within the *Anastrepha fraterculus* complex, the Andean morphotype is allopatric, while the Brazilian morphotypes (Brazilin-1, Brazilian-2 and Brazilian-3) are sympatric (Hernández-Ortiz et al. 2012, Selivon et al. 2005). The sympatry of the Brazilian entities may be one of the possible factors contributing to the similarity of their chemical profiles. Evidence of similarities of pheromone profiles within and between the species complexes of sympatric populations comes from an extensive evolutionary study of Hawaiian Drosophilidae CHs (Alves et al. 2010). Such evolutionary studies are missing for the *Anastrepha fraterculus* complex and nothing is known about the role of pheromones (short- or long-range) in the speciation process of this species.

The Andean morphotype populations had very different pheromone profiles from that of the two Brazilian morphotypes. Břízová et al. (2013) and Cáceres et al. (2009) reported extensive qualitative and quantitative differences in the male pheromone composition of Brazilian, Argentinean and Peruvian populations of the *Anastrepha fraterculus* complex. Our study demonstrated that the common plant monoterpene, limonene, varies least among all populations. Two of the most variable volatiles from the male pheromone mixture were (*E*, *Z*)-3,6-nonadien-1-ol and (*E*, *E*)-α-farnesene. As reported by Milet-Pinheiro et al. (2015), (*E*, *Z*)-3,6-nonadien-1-ol, α-farnesene, and epianastrephin are highly attractive to conspecific females after approaching a mating site, while plant compounds such as α-pinene or limonene are used by female to find mating and brood sites (Robacker and Hart 1986). We speculate that the different ratios of (*E*, *Z*)-3,6-nonadien-1-ol, (*E*, *E*)-α-farnesene, and epianastrephin, together with the diverse CH profiles, result in the final rejection of a male by an heterospecific female. A recent study on reproductive compatibility demonstrated that the Andean morphotype is fully incompatible with the Brazilian-1 and Brazilian-3 morphotypes (Devescovi et al. 2014). The authors stated that prezygotic sexual incompatibility might be a result of the differences in the timing of mating activities between the morphotypes studied. Considering the mating of *Anastrepha fraterculus*, where chemical communication plays an important role in acceptance or rejection of males, we suggest that the differences in the chemical profiles identified in the present study may also contribute to sexual incompatibility. Nevertheless, further electrophysiological and behavioural studies involving different morphotypes of the *Anastrepha fraterculus* complex need to be performed in order to evaluate this hypothesis.

Male and female flies of the Andean morphotype and the Brazilian morphotypes can be separated using CH profiles (Vaníčková et al. in press). The Andean morphotype formes a separated group whereas the Brazilian-1 and Brazilian-3 morphotypes create a monophyletic cluster. Nonetheless, Brazilian populations belonging to the same geographical areas do not group together. Variation in the chemical composition of CH profiles identified here may be influenced by genetic variability within and between populations of the Brazilian-1 and 3 morphotypes. It is important to note that all of the Brazilian populations investigated here were created during several generations under identical laboratory conditions, which could possibly influence the results presented here. Houot et al. (2010) reported the effects of laboratory acclimation on the variation of male courtship, mating and the production of sex pheromone, in *Drosophila melanogaster*. These authors concluded that the reproduction-related characters could diverge between neighboring *Drosophila melanogaster* populations, and differently adapt to stable laboratory conditions. Nevertheless, in tephritidae these kinds of studies are missing.

Selivon et al. (2004) found conspicuous differences between sex chromosomes that separated the Brazilian-3 from the Brazilian-1 and Brazilian-2 morphotypes. The possibility for hybridization between distinct cryptic species within the *Anastrepha fraterculus* complex and meiotic recombination of chromosomal markers could form the genetic basis by which CHs vary between related putative species. The evidence comes from experiments with *Drosophila* spp. that explained interspecific variation of CH profiles (Coyne et al. 1994, Coyne and Oyama 1995, Doi et al. 1996, Coyne and Charlesworth 1997). Future genetic analyses are necessary for evaluation of this hypothesis within the *Anastrepha fraterculus* complex.

CHs in insects serve primarily to prevent desiccation by reducing water loss (Blomquist et al. 1987, Blomquist and Bagnères 2010). Populations living in warmer and drier environments lose water less rapidly and usually have longer chain-length CHs than populations in humid habitats (Blomquist and Bagnères 2010). However, the relationship between CH structure and the capacity to resist desiccation is not so simple. In our previous work on CH profiles from geographically distinct populations of the *Anastrepha fraterculus* complex, we reported that the relative proportions of these compounds vary, depending on relative humidity, relative temperature and altitude (Vaníčková et al. in press). In the present study, CH profiles from the Andean morphotype formed one single cluster. These populations live naturally at high altitudes with lower relative temperatures, and therefore their specific CHs are long methylbranched compounds (e.g. methylheptatriacontane). However, the same conclusion is not possible to draw for the Brazilian-1 and Brazilian-3 morphotypes studied. The three Brazilian morphotypes may co-occur in the same localities infesting guava fruits (*Psidium guajava* L.) (Selivon et al. 2004). The Brazilian-3 morphotype seems to be restricted to the Atlantic coastal areas and may co-occur with the Brazilian-2 morphotype infesting guava and tropical almond (*Terminalia catappa* L.). The Brazilian-1 morphotype occurs from northern Argentina through southern and southeastern Brazil and may also co-occur together with the Brazilian-2 morphotype infesting guavas and oranges (*Citrus* sp.). In Brazil there are 70 different host plants for the *Anastrepha fraterculus* complex recorded (Zucchi 2007). Etges and Jackson (2001) reported that the variation of CH profiles between closely related species or between populations of these species of *Drosophila mojavensis*, reflects the adaptation to different host plants. In these flies, the ratio of the principal CHs rapidly changed with laboratory acclimation, and influenced courtship and mating (Stennett and Etges 1997, Houot et al. 2010). These CH changes depend on enzymes whose level could represent a metabolic adaptation to host-plant chemicals (Higa and Fuyama 1993, Jones 2001, Houot et al. 2010). Varying the availability of different nutrients could also account for CH variation between strains raised in the laboratory (Stennett and Etges 1997). These factors, possibly influencing CH composition in the *Anastrepha fraterculus* complex, need to be carefully investigated in future studies.

## Conclusion

The present study demonstrates that pheromone components and CH profiles diverge qualitatively between Andean and Brazilian-1 and Brazilian-3 morphotypes and may be used to some extent to delimit morphotypes in the *Anastrepha fraterculus* species complex. Comprehensive studies, which simultaneously examine environmental, behavioural, genetic and chemical features are necessary to be performed aiming to understand which factors affect the geographical variation in the male-borne volatiles and CH profiles in the *Anastrepha fraterculus* complex.

## Supplementary materials

Supplementary material 1 Table S1. *Anastrepha fraterculus* male and female characteristic. Data type: species data Explanation note: *Anastrepha fraterculus* male (m) and female (f) characteristic cuticular hydrocarbons identified by principal component analyses. This dataset is made available under the Open Database License (http://opendatacommons.org/licenses/odbl/1.0/). The Open Database License (ODbL) is a license agreement intended to allow users to freely share, modify, and use this Dataset while maintaining this same freedom for others, provided that the original source and author(s) are credited.Lucie Vaníčková, Radka Břízová, Antonio Pompeiano, Luana Lima Ferreira, Nathaly Costa de Aquino, Raphael de Farias Tavares, Laura D. Rodriguez, Adriana de Lima Mendonça, Nelson Augusto Canal, Ruth Rufino do Nascimento
