# Supplementary material for: Characterisation of the chemical profiles of Brazilian and Andean morphotypes belonging to the Anastrepha fraterculus complex (Diptera, Tephritidae)
Source: Zookeys. 2015 Nov 26;(540):193–209. doi: 10.3897/zookeys.540.9649 (PMC4714070; doi:10.3897/zookeys.540.9649)
Supplement: Supplementary material 1 — Table S1. Anastrepha fraterculus male and female characteristic. [file zookeys-540-193-s001.docx]

|  |  | Andean morph. |  |  | Brazilan-1 morph. |  |  | Brazilian-3 morph. |
| --- | --- | --- | --- | --- | --- | --- | --- | --- |
| Abbreviation^‡^ | Compound | CAC population | DUI population | SIB population | BEN population | SAO population | PEL population | AL population |
| A1 | *n*-C12 | f | f | f |  |  |  |  |
| A7 | *n*-C22 |  |  |  | f | f | f | f |
| A10 | *n*-C25 | f | f | f |  |  |  |  |
| A14 | *n*-C29 |  |  |  | m | m | m | m |
| A16 | *n*-C31 | f | f | f | m | m | m | m |
| B3 | 3-MeC27 |  |  |  | m | m | m | m |
| B4 | 2-MeC28 |  |  |  | f | f | f | f |
| B5 | 9-/11-/13-MeC29 | m | m | m |  |  |  |  |
| B7 | 3-MeC29 |  |  |  | f | f | f | f |
| B9 | 9-/11-/13-MeC31 | f | f | f |  |  |  |  |
| B11 | MeC37 |  |  |  | m | m | m | m |
| C8 | C31:1 |  |  |  | f | f | f | f |
| C10 | 11-C33:1 |  |  |  | m | m | m | m |
| D4 | C34:2 |  |  |  | f | f | f | f |
| D7 | C37:2 | f | f | f |  |  |  |  |

Appendix. *Anastrepha fraterculus* male (m) and female (f) characteristic cuticular hydrocarbons identified by principal component analyses.

^‡^Abbreviation corresponds to Figures 3 and 4.
